# Protein Bioinformatics Infrastructure for the Integration and Analysis of Multiple High-Throughput “omics” Data

**DOI:** 10.1155/2010/423589

**Published:** 2010-03-29

**Authors:** Chuming Chen, Peter B. McGarvey, Hongzhan Huang, Cathy H. Wu

**Affiliations:** ^1^Department of Computer & Information Sciences, Center for Bioinformatics & Computational Biology, University of Delaware, Newark, DE 19711, USA; ^2^Protein Information Resource, Department of Biochemistry and Molecular & Cellular Biology, Georgetown University Medical Center, Washington, DC 20007, USA

## Abstract

High-throughput “omics” technologies bring new opportunities for biological and biomedical researchers to ask complex questions and gain new scientific insights. However, the voluminous, complex, and context-dependent data being maintained in heterogeneous and distributed environments plus the lack of well-defined data standard and standardized nomenclature imposes a major challenge which requires advanced computational methods and bioinformatics infrastructures for integration, mining, visualization, and comparative analysis to facilitate data-driven hypothesis generation and biological knowledge discovery. In this paper, we present the challenges in high-throughput “omics” data integration and analysis, introduce a protein-centric approach for systems integration of large and heterogeneous high-throughput “omics” data including microarray, mass spectrometry, protein sequence, protein structure, and protein interaction data, and use scientific case study to illustrate how one can use varied “omics” data from different laboratories to make useful connections that could lead to new biological knowledge.

## 1. Introduction

Unlike traditional one-gene-at-a-time approach, which provides the detailed molecular functions of individual genes, the advances of high-throughput technologies in the study of molecular biology systems in the past decades marked the beginning of a new era of biological and biomedical research, in which researchers systematically study organisms on the levels of genomes (complete genetic sequences) [1], transcriptomes (gene expressions) [2], proteomes (protein expressions) [3], metabolomes (metabolic networks) [4], and interactomes (protein-protein interactions) [5]. 

Genomics analysis tells us the complete genetic sequences and the intragenomic interactions within the genomes. The sequences only tell us what a cell can potentially do. In order to know what a cell is doing, DNA microarray technologies [6] have been used to study the transcriptomes, also called Gene Expression Profiling [7], which examines the expression level of mRNAs of thousands of genes to give a global view of the cell functions under various conditions. Recently, high-throughput gene expression profiling technologies have been applied to help biomarker discovery and identification of molecular targets related to human cancer [8].

The genome of an organism is relatively constant, while the proteome of an organism, a set of expressed proteins under varied conditions, can be quite different for different cell types and conditions. Because the expression profiling at the transcript level can only give a rough estimate of the concentration of expressed proteins, high-throughput profiling at the protein level using mass spectrometry technologies has been widely used to identify, characterize, and quantify all the proteins and their functions in cells under a variety of conditions [9, 10]. Since most physiological and pathological processes are manifested at the protein level, biological scientists are growingly interested in applying the proteomics techniques to foster a better understanding of basic molecular biology, disease processes, and discovery of new diagnostic, prognostic, and therapeutic targets for numerous diseases [11, 12]. Metabolic profiling [13], which involves the chemical process of metabolites, can show the physiology of cells at a given time; together with the expression profiling at the transcript and protein levels, they can give a fairly complete view of living organisms [14–16].

The rapid growth of high-throughput genomics, proteomics, and other large-scale “omics” data presents both opportunities and challenges in terms of data integration and analysis. Many bioinformatics databases and repositories have been developed to organize and provide biological annotations for individual genes and proteins to facilitate the sequence, structural, functional, and evolutionary analyses of genes and proteins in the context of pathway, network, and systems biology. In addition, a rapidly growing number of quantitative methods and tools have been developed to enable efficient use and management of various types of “omics” data and analyses of large data sets for different biological problems, including biomarker discovery for diagnosis and early detection of disease. A few examples include (1) Bioconductor [17] for gene expression analysis; (2) TranscriptomeBrowser [18] for data mining of publicly available microarray data; (3) SEQUEST [19], Mascot [20], and X! Tandem [21] for protein identification, quantification, and characterization; (4) Trans-Proteomic Pipeline [22, 23] for uniform analysis of LC-MS/MS proteomics data; (5) MetaboMiner [24] for semi-automatic identification of metabolites in complex biofluids from 2D NMR spectra; (6) APID [25] for integration of main known experimentally validated protein-protein interactions. 

The richness of “omics” data allows researchers to ask complex biological questions and gain new scientific insights. However, the voluminous, complex, and context-dependent data being maintained in heterogeneous and distributed environments plus the lack of well-defined data standard, and standardized nomenclature imposes a major challenge for all parties involved, from lab technicians, data analysts to biomedical researchers who are trying to interpret the final results of “omics” experiments. Therefore, advanced computational methods and bioinformatics infrastructures are needed for integration, mining, visualization, and comparative analysis of multiple high-throughput “omics” data to facilitate data-driven hypothesis generation and biological knowledge discovery.

In this paper, we present the challenges in high-throughput “omics” data integration and analysis in Section 2, introduce a protein-centric approach for systems integration of large and heterogeneous high-throughput “omics” data including microarray, mass spectrometry, protein sequence, protein structure and protein interaction data in Section 3, and use NIAID (National Institute of Allergy and Infectious Diseases) Biodefense Proteomics Resource as a case study to illustrate how one can use varied “omics” data from different laboratories to make useful connections that could lead to new biological knowledge in Section 4. We conclude this paper and present future work in Section 5.

## 2. Challenges in “omics” Data Integration and Analysis

### 2.1. Data Heterogeneity and Complexity

The most commonly used molecular biology databases for functional analysis of gene and protein expression data are listed in Table 1. They fall into the following categories: protein sequence, gene and genome, taxonomy, gene expression, protein peptide ID databases, protein expression, function and pathway, genetic variation and disease, ontology, interaction, modification, structure, and classification. The heterogeneity and complexity of data in those databases are due to the different attributes of genes and proteins and the context of data processing and analysis, that is, sequence, structure, function and pathway, and so forth. The unique structures of these molecular biology databases reflect the different underlying biological models. Most of the databases provide search, browse, and data download functionalities, some of them also provide a set of analysis tools. In order to use these databases effectively, one must understand the database schemas in different data sources and their relationships. Data sources often contain overlapping or similar data elements, such as database identifiers, organism names, protein names, and sequences, which are the keys to connecting them. However, there may be conflicting data definitions among the data sources. Therefore, bioinformatics tools are needed to uncover the relationships among the databases and to map biological entities from one database to another.

### 2.2. Data Provenance and Biological Knowledge

In many cases, one of the most difficult tasks is not mapping biological entities from different sources or managing and processing large set of experimental data, such as raw microarray data, 2D gel images, and mass spectra. The problem was recording the detailed provenance of those data, that is, what was done, why it was done, where it was done, which instrument was used, what settings were used, how it was done, and so forth. The provenance of experimental data is an important aspect of scientific best practice and is central to the scientific discovery [67]. Although great efforts have been put to develop and maintain data format standards, for example, mzXML [68], HUPO PSI (HUPO Proteomics Standards Initiative) [69], MAGE-TAB (Spreadsheet-based format for Microarray data) [70], MAGE-ML (Microarray Gene Expression Makeup Language) [71], and so forth, and minimal information standards describing such data, for example, MIGS (Minimum Information about a Genome Sequence) [72], MIAME (Minimum Information About a Microarray Experiment) [73], MIAPE (Minimum Information About a Proteomics Experiment) [74], and MIAMET (Minimum Information About a Metabolomics Experiment) [75], the ontologies and related tools which provide formal representation of a set of concepts and their relationships within the domain of “omics” experiments still lag behind the current development of experimental protocols and methods. The standardization of data provenance remains a somewhat manual process, which depends on the efforts of database maintainers and data submitters.

The general biomedical scientists are more interested in finding and viewing the “knowledge” contained in an already analyzed data set. However, in high-throughput research many of the gene/protein data generated are insignificant in the conclusions of an analysis. Of the thousands of genes examined in a microarray experiment, only a relatively few show significant responses relevant to the treatment or condition under study. Unfortunately, this information seldom comes with the standard data files and formats and is usually not easily found in “omics” repositories unless a re-analysis is performed or the data is annotated by a curator. For example, tables of proteins present in a given proteomics experiment or genes found to be up- or down-regulated under defined conditions are routinely found as supplemental data in scientific publications but are not available in a searchable or easily computable form anywhere else. This is unfortunate as this supplemental information is the result of considerable analysis by the original authors of a study to minimize false positive and false negative results and often represents the “knowledge” that underlies additional analysis and conclusions reached in a publication.

### 2.3. Comparative Analysis

Recently, “omics” data analysis has focused on information integration of multiple studies including cross-platform, cross-species, or cross-disease-type analyses [76–82]. There are urgent needs for developing computational methods to integrate data from multiple studies and to answer more complex biological questions that traditional methods cannot provide. Comparing experimental results across multiple laboratories and data types can also help forming new hypotheses for further experimentation [83–85]. Different laboratories use different experimental protocols, instruments, and analysis techniques, which make direct comparisons of their experimental results difficult. However, having related data in one place can make queries and comparisons of combined protein and gene data sets and further analysis possible.

## 3. Protein-centric “omics” Data Integration and Analysis

### 3.1. Data Integration

As the volume and diversity of data and the desire to share those data increase, we inevitably encounter the problem of combining heterogeneous data generated from many different but related sources and providing the users with a unified view of this combined data set. This problem emerges in the life sciences research community, where research data from different bioinformatics data repositories and laboratories need to be combined and analyzed. The benefit of developing a data integration system is that it can facilitate information access and reuse by providing a common access point. It also provides users with more complete view of the available information. 

Lenzerini [86] proposed a logical framework for data integration systems from a theoretical perspective based on the notion of global schema, where the goal of data integration system is to provide the users with a homogeneous view of the data across different sources. In this theoretical model, data integration can be characterized into two approaches: LAV (Local-As-View) versus GAV (Global-As-View). The LAV approach is the most effective approach when the global schema is stable in the data integration system. The example of this approach is *data warehouse*. The data warehouse approach put data sources into a centralized location with a global data schema and an indexing system for fast data retrieval. The GAV approach is the most effective approach when the set of sources are stable in the data integration system. The example of this approach is *federated database*. The federated database approach does not require a centralized database. It maintains a common data model and relies on a schema mapping to translate heterogeneous database schema into the target schema for integration. Therefore, it is modular, flexible, and scalable.

In our experience, the users of microarray, proteomics and, other “omics” data can be broadly divided into two groups: (1) bioinformaticians or biostatisticians who develop tools to handle large and complex data set routinely; (2) general biomedical scientists who lack the expertise or tools to do “omics” data analysis but still want to analyze the data sets and find the biological knowledge related to the set of genes or proteins they are studying. Considering such target user groups, our approach for integration of diverse high-throughput “omics” data is to construct a relatively lightweight data warehouse to capture the key information or “knowledge” our users are likely to need. 

In our approach, the original data may reside in other databases or repositories that are managed and optimized for a particular type of “omics” data such as microarray and mass spectrometry data. Our warehouse uses Web Services, database downloads and other means to make updates regularly with web links back to the original data sources. Our approach uses less computational resources and human involvement, it meanwhile provides the usability, flexibility, reliability and performance. As proteins occupy a middle ground molecularly between gene and transcript information and higher levels of molecular and cellular structure and organization, the key design principle of our data integration approach is to integrate diverse “omics” data and present them in a protein-centric fashion where information query is conducted via common proteins and their large set of attributes such as families, functions, and pathways.

### 3.2. Protein ID Mapping Service

The use of different data sources and identifiers in analysis pipelines is a common problem encountered when we try to combine the data across multiple laboratories or research centers. One of the most difficult problems in “omics” data integration and analysis is to maintain the correspondence of IDs for genes and proteins and their high-level functional attributes such as modifications, pathways, structures, and interactions. The ID or name mapping [87–89] can provide a comprehensive means to connect different data sets and serves as a key search criterion for users to search any gene or protein of their interests.

**Figure 1:**
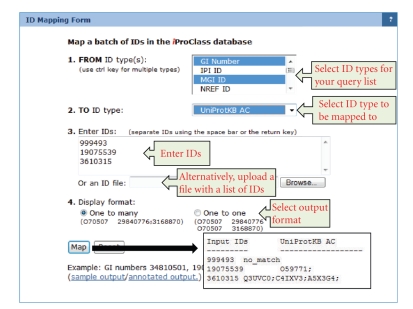
PIR ID mapping service maps a set of NCBI GI numbers to UniProt accession numbers.

The Protein Information Resource (PIR) provides an ID mapping service (http://ProteinInformationResource.org/pirwww/search/idmapping.shtml) (Figure 1) that maps between UniProtKB and other data sources (Table 2) to support data interoperability among disparate data sources and to allow integration and query of data from heterogeneous molecular biology databases. Some mappings are inherited from cross-references within UniProtKB entries, some are based on the existing bridge between EMBL and GenBank entries, and others make use of cross-references obtained from the iProClass database (see Section 3.3). A subset of the latter (such as between UniProtKB accession number and NCBI GI number) requires matching based on sequence and taxonomy identity. Thus, it is possible to map between numerous databases using only a few sources for the mapping itself. The PIR ID mapping service focuses on two types of ID mapping [90]: (1) mapping among the biological objects, for example, mapping between NCBI GI number and UniProtKB accession number is a protein-to-protein mapping; (2) mapping from biological objects to their attributes, such as mappings from protein accession numbers to GO IDs.

### 3.3. iProClass Data Warehouse

PIR provides iProClass (http://ProteinInformationResource.org/pirwww/dbinfo/iproclass.shtml) [91, 92], a data warehouse for fast retrieval of voluminous, heterogeneous, and distributed “omics” data and serves as the central protein-centric infrastructure. iProClass is currently built around the UniProtKB [26] and supplemented with additional sequences from gene models in RefSeq [27] and Ensembl [93] and additional annotation and literature from other curated data resources such as Model Organism Databases (http://gmod.org/wiki/Main_Page) and GeneRIF [94]. Coupled with the PIRSF protein family classification system [62, 95], the data integration in iProClass reveals interesting relationships among protein sequence, structure and function, and facilitates functional analysis in a systems biology context. 

**Figure 2:**
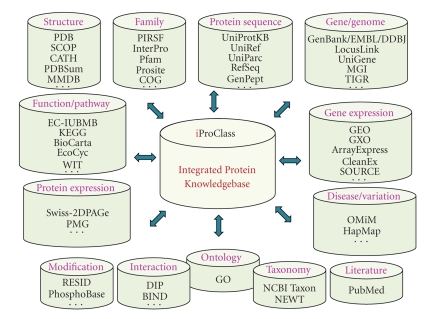
The overview of PIR iProClass data warehouse.

iProClass database contains full descriptions of all known proteins with up-to-date information from many sources (Figure 2), thereby providing much richer annotation than can be found in any other single database [92]. The current version of the iProClass database provides value-added report for about 10 million protein entries, including all entries in the UniProtKB and unique NCBI entries. It provides rich links and executive summaries from more than 100 databases (http://ProteinInformationResource.org/cgi-bin/iproclass_stat) of protein sequences, families, functions, and pathways, protein-protein interactions, post-translational modifications, structures and structural classifications, genes and genomes, ontology, literature, and taxonomy. Source attribution and hyper-text links facilitate the exploration of additional information and the examination of discrepant annotations from different sources. 

iProClass database is implemented in Oracle and updated every three weeks. The underlying database schema and update procedures have been modified to interoperate with UniProtKB. iProClass also provides comprehensive views for more than 35,000 PIRSF protein families [62]. PIRSF families are curated systematically based on literature review and integrative sequence and functional analysis, including sequence and structure similarity, domain architecture, functional association, genome context, and phylogenetic pattern. The results of classification and expert annotation are summarized in PIRSF family reports, with graphical viewers for taxonomic distribution, domain architecture, family hierarchy, multiple alignment, and phylogenetic tree [95]. The integrative data warehouse approach like iProClass has led to novel prediction and functional inference for uncharacterized proteins, allows systematic detection of genome annotation errors, assists comparative studies of protein function and evolution [95], and provides sensible propagation and standardization of protein annotations [96, 97].

iProClass provides a set of data search and retrieval interfaces and value-added views for UniProtKB protein entries and PIRSF family entries with extensive annotations and graphical display of reported information. 

#### 3.3.1. Entry Retrieval

**Figure 3:**
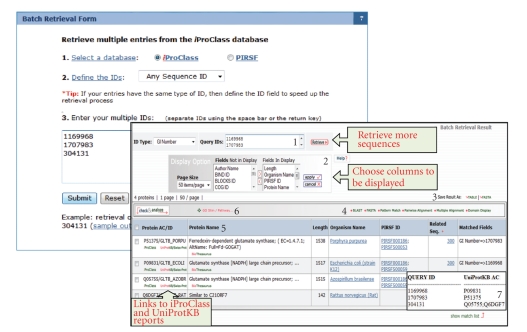
iProClass data warehouse batch retrieval tool web form and result page. (1)* Retrieval Box*: it shows the user's query ID and also allows the user to perform a new retrieval; (2) *Display Options*: it allows the user to choose the columns to be displayed; (3) *Save Results As*: the output can be saved to the user's local computer. The results will be saved for selected entries or, if no proteins are selected, for all entries; (4) *Analyze*: BLAST, FASTA, Pattern Match, Multiple Alignment, and Domain Display: retrieved entries can be further analyzed using the sequence analysis programs available on the results page; (5) *Results Display*: search results are displayed in a table; (6) *GO Slim*: it shows smaller versions of the Gene Ontologies (GO) containing a subset of the terms in the whole GO. They give a broad overview of the ontology content without the detail of the specific fine grained terms; (7) *Show match list*: it shows a table mapping the user's query IDs with the UniProtKB/UniParc IDs.

The iProClass website provides a very simple way to retrieve protein entries by a single protein ID or one of many other sequence database identifiers. It also allows retrieval of protein entries using a batch of database identifiers. The batch retrieval tool (http://ProteinInformationResource.org/pirwww/search/batch.shtml) (Figure 3) provides high flexibility, allowing the retrieval of multiple entries from the iProClass database by selecting a specific identifier or a combination of them. Batch retrieval of PIRSF families using a subset of these identifiers can also be done as well. 

#### 3.3.2. Peptide Match

**Figure 4:**
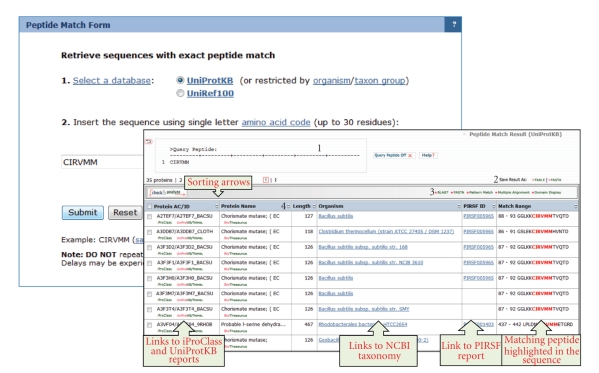
iProClass data warehouse peptide match tool web form and result page. (1) *Query Peptide*: it displays ON/OFF the query peptide by clicking this box; (2) *Save Results As*: the output can be saved to the user's local computer. The results will be saved for selected entries or, if no proteins are selected, for all entries; (3) *Analyze*: BLAST, FASTA, Pattern Match, Multiple Alignment, and Domain Display: retrieved entries can be further analyzed using the sequence analysis programs available on the results page; (4) *Results Display*: search results are displayed in a table.

Peptide sequences, such as those obtained by MS/MS proteomics experiments, can be used as queries to search proteins containing exact matches to the peptide sequence from the UniProtKB database. In this case, the search can be performed on the whole set of proteins or on only those from Taxonomy group or a specific organism, as in the example shown in Figure 4. Peptide Match tool (http://ProteinInformationResource.org/pirwww/search/peptide.shtml) may reveal protein sequence regions that are completely conserved in a certain group of organisms and that could be important for functions of a protein.

#### 3.3.3. Summary Report

iProClass integrated database provides two types of summary report for the information presentation: Protein summary report and Family summary report. The Protein summary report contains information about protein ID and name, source organism taxonomy, sequence annotations, data cross-references, family classification, and graphical display of domains and motifs on the amino acid sequence. A sample Protein summary report can be viewed here (http://ProteinInformationResource.org/cgi-bin/ipcEntry?id=P04637). The Family summary report is only available for PIRSF families and contains information about PIRSF number and general statistics, family and function/structure relationships, database cross-references, and graphical display of domain and motif architecture of seed members or all members. A sample Family summary report can be viewed here (http://ProteinInformationResource.org/cgi-bin/ipcSF?id=PIRSF000186).

## 4. Integrative Analysis of Multiple High-Throughput “omics” Data

In this section, we use the NIAID (National Institute of Allergy and Infectious Diseases) Biodefense Proteomics Resource (http://ProteinInformationResource.org/pirwww/proteomics/ ) as a case study to briefly demonstrate how our protein-centric approach can be applied to integrate and support mining and analysis of large and heterogeneous high-throughput “omics” data. The architecture and detailed features of the Biodefense Proteomics Resource have been described elsewhere [98, 100].

### 4.1. Data Sources

The NIAID Biodefense program consists of seven Proteomics Research Centers (PRCs) conducting state-of-the-art high-throughput research on pathogens of concern in biodefense and emerging infectious diseases as well as a Biodefense Resource Center for public dissemination of the pathogen and host data, biological reagents, protocols, and other project deliverables (Table 3). The PRCs work on many different organisms, covering bacterial pathogens and viral pathogens. The centers generated a heterogeneous set of experimental data using various technologies loosely defined as proteomic, but encompassing genomic, structural, immunology, and protein interaction technologies, as well as more standard cell and molecular biology techniques used to validate potential targets identified via high-throughput methods. In addition to the data, the PRCs have provided biological reagents such as clones, antibodies, and engineered bacterial strains; other deliverables include standard operating procedures (SOPs) and new technologies such as instrumental methods and software tools and finally publications related to all of these activities.

### 4.2. Master Protein Directory

Based on the functional requirements of the Resource Centers, we developed a protein-centric bioinformatics infrastructure for integration of diverse data sets. Multiple data types from PRCs are submitted to the center using a data submission protocol and standard exchange format, with the metadata using controlled vocabulary whenever possible. Underlying the protein-centric data integration is a data warehouse called the Master Protein Directory (MPD) [98] where key information is extracted from the primary data stored in the Proteomics Data Center, and combined for rapid search, display, and analysis capabilities. The MPD is built on the data and capabilities of iProClass data warehouse. Currently the MPD defines and supports information from the following types of data produced by the PRCs: mass spectrometry, microarray, clones, protein interaction, and protein structure [98]. 

The MPD focused on capturing significant results usually only available in supplementary tables for the primary authors. To enable searching on these results, it needs to be converted into a searchable and digested form and mapped to the gene or protein of interest. To achieve this goal we developed a simple set of defined fields called *“structured assertions”* that could be used across proteomics, microarray, and possibly other data types [98]. A *“structured assertion”* can represent the results in a simple form like “Protein *V* (presented) in experimental condition *W*,” where *V* represents any valid identifier and *W* represents values in a simple experimental ontology. We implemented a simple two-field assertion for the analyzed results of proteomics and microarray data and “experimental condition” field containing simple keywords to describe the key experimental variables (growth conditions, sample fractionation, time, temperature, infection status, and others) and “Expression Status” which has three options: increase, decrease or present. Though seemingly simple, the approach provides unique analytical power in the form of enabling simple queries across results from different data types and laboratories.

### 4.3. Integrated Discovery Platform

We have developed methods and prototype software tools specifically designed to provide functional and pathway discovery of large-scale “omics” data in a systems biology context with rich functional descriptions for individual proteins and detecting functional relationships among them. A prototype expression analysis system, integrated Protein eXpression (iProXpress) (http://ProteinInformationResource.org/iproxpress) [90, 101], was recently developed and has been applied to several studies [102–104]. The iProXpress system consists of several components, including a data warehouse composed of the UniProtKB and iProClass databases, and analysis tools for protein mapping, functional annotation, and expression profiling. Sequence homology analysis tools are also included in the protein mapping tools. System integration by iProXpress also supports iterative functional analysis. The major functionalities provided by the iProXpress system include the mapping of gene/protein sequences with different types of IDs from gene expression and proteomic data to UniProtKB protein entries as described in Section 3.2 and the functional annotation and profiling of the mapped proteins for functional analysis in a systems biology context.

#### 4.3.1. Functional Annotation

After the ID mapping of proteins, rich annotation can be fully described in a protein information matrix based on sequence analysis and integration of information from the MPD. We precompute and regularly update sequence features of functional significance for UniProt proteins, and make the sequence analysis tools available for online analysis of proteins/sequence variations not in UniProt database. Sequence features precomputed include homologous proteins in KEGG [47], BioCarta (http://www.biocarta.com/), and other curated pathway databases to populate pathway annotation, InterProScan [105] for family, domain and motif identification, and Phobius [106] for transmembrane helix and signal peptide prediction. Properties derived from homology-based inference are presented in the information matrix with evidence attribution.

#### 4.3.2. Functional Profiling

Functional profiling analysis aims at discovering the functional significance of expressed proteins, the plausible functions and pathways, and the hidden relationships and interconnecting components of proteins, such as proteins sharing common functions, pathways, or cellular networks. The extensive annotation in the protein information matrix allows functional categorization and detailed analysis of expressed proteins in a given data set as well as cross-comparison of co-expressed or differentially-expressed proteins from multiple data sets. For functional categorization, proteins are grouped based on annotations such as GO [51] terms, and KEGG [47] and BioCarta pathways, and then correlated with sequence similarity to identify relationships among individual proteins or protein groups. The functional categorization chart displays the frequency (number of occurrences) of proteins in each functional category. Categorization and sorting of proteins based on functions, pathways, and/or other attributes in the information matrix generate various protein clusters, from which interesting unique or common proteins in different data sets can be identified in combination with manual examination. The cross-comparison matrix shows the comparative distribution of functional categories in multiple data sets.

### 4.4. Data Mining and Analysis

In the NIAID proteomics resource center project, our support for data mining and analysis was designed to make sure that all project data and other deliverables are available via browsing and simple keyword search; the data and information are sufficient for re-analysis or mining by a skilled researcher; the data, procedures, publications, and general results and conclusions of an analysis are easily searchable for a biomedical scientist who is not familiar with the details of the particular technologies used to generate them. We focused on providing simple, yet powerful, queries of experimental summaries where a user can query if a gene/protein was presented in the results. Once a set of proteins of interest is identified, user can further view the specific experimental values, methods used to generate the particular data set, and all protein attributes such as protein names, accessions, or project data, and search pathways, protein families, Gene Ontology (GO) [51] terms, and database cross-references, and so forth.

**Figure 5:**
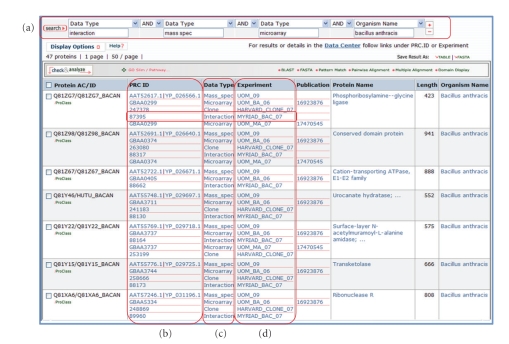
Protein-centric query across multiple data types in the NIAID Biodefense Master Protein Directory. (a) Search for *Bacillus anthracis* proteins with data from interaction, microarray and mass spectrometry data yields 47 bacterial proteins; (b) ID mapping merges database identifiers from up to six different databases; (c) Different data types are displayed; (d) Each data set is assigned an experiment identifier and hyperlinks provide additional information about the experiments.

**Figure 6:**
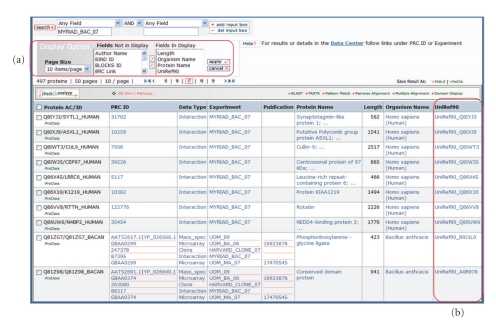
A single experiment, Myriad_Bac_07, contains interactions between 497 *Bacillus anthracis* and human proteins determined by Yeast two Hybrid analysis. Using the customizable interface we can download the UniRef_90 [99] identifiers for human proteins and use them to retrieve the homologous mouse proteins with microarray and mass spectrometry data from mouse macrophages infected with Bacillus anthracis. See text for details. (a) Customizable interface can be used to display UniRef_90 identifiers of proteins; (b) A list of human proteins used to retrieve the homologous mouse proteins with microarray and mass spectrometry data from mouse macrophages infected with Bacillus anthracis.

**Figure 7:**
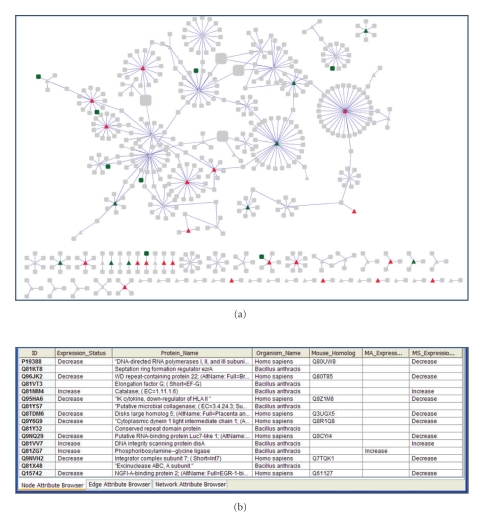
Pathogen-Host Y2H protein interaction data, from Figures 5 and 6, was loaded into Cytoscape and combined with microarray and mass spectrometry data from other experiments where Bacillus anthracis was used to infect mouse macrophages. Only data that showed a significant increase or decrease in expression in the original experiments was loaded. For human interacting proteins, data from homologous mouse proteins was used. (a) The Bacillus anthracis—human protein interaction network. Triangles are Bacillus anthracis proteins, and squares are human proteins. Red indicates that an increase in expression was observed in either microarray or proteomic experiment, and green indicates a decrease in expression; (b) A list of sixteen proteins involved in eight pathogen-host interactions where a human protein showed a significant decrease in expression upon infection. Of the eight interactions three were with pathogen proteins that showed an increase in expression upon infection.

The MPD web interface with its ability to mine the data and download information to other analysis tools has been used to identify and rank potential targets for therapeutics and diagnostics [98]. An example is also shown in Figures 5, 6, and 7. Figure 5 shows a query for *Bacillus anthracis *with microarray, mass spectrometry, and interaction data. 47 proteins met the criteria. The protein centric ID mapping service helps make this combination possible as each research center used different protein IDs for their works and in some cases multiple IDs for the same protein. 

Inspection of the protein interaction data showed that it contained a total of 84 bacterial proteins interacting with 412 Human proteins (Figure 6). However all the host microarray and mass spectrometry data in the MPD come from experiments in a mouse macrophage model. The comprehensive protein warehouse allows us to find the human-mouse homologs via family classifications or sequence clustering and thus allows us to combine and view the interaction data with microarray and mass spectrometry data. 

We downloaded the UniRef_90 [99] cluster ID for each interacting human host protein and retrieved all related mouse proteins data in the MPD. UniRef_90 clusters all UniProtKB sequences at 90% sequence identity with no gaps and thus provides a quick and easy way to find closely related proteins. We downloaded the interaction data, mass spectrometry data, and microarray data for *Bacillus anthracis, *Human and Mouse, combined and visualized them using Cytoscape [107]. Figure 7 shows the resulting network of pathogen and host proteins with proteins that have increased or decreased expression in response to infection (detected by microarray and/or mass spectrometry experiments) highlighted in color. Analyses like these can help highlighting proteins for further analysis. For example, Figure 7 reveals that eight host proteins that decreased in abundance on infection also interact with eight *Bacillus anthracis *proteins. Three of the eight interacting *Bacillus anthracis *proteins showed an increase in expression on infection. The combination of significant expression changes and interaction data between pathogen and host suggests that these interactions maybe real and of importance to infection and virulence and should be prioritized for further study.

## 5. Conclusions and Future Work

The availability of voluminous, complex, and context-dependent high-throughput “omics” data brings both challenges and opportunities for bioinformatics research. The integrative analysis across multiple data sets can reveal the potential functional significance and hidden relationships between different biological entities, which requires advanced computational methods and bioinformatics infrastructures to support integration, mining, visualization, and comparative analysis to facilitate data-driven hypothesis generation and biological knowledge discovery.

Our protein-centric integration approach based on Protein ID mapping service, iProClass data warehouse, and iProXpress discovery platform provides a simple but powerful bioinformatics infrastructure for scientific discovery and hypothesis generation. The case study using NIAID Biodefense Proteomics Resource as an example illustrates that our protein-centric data integration allows query and analysis across different data types and pathogen host systems that lead to new biological knowledge. It is also a relatively simple, yet powerful and practical, approach to integrate and navigate diverse sets of “omics” data in a manner useful for systems biology study.

As the future work, the prototype system iProXpress will be further developed into a pipelined analysis tool to allow direct integration of multiple high-throughput “omics” experimental results. Moreover, the network modeling method will also be incorporated for functional and pathway analysis in a broader range of biological systems. We will also explore the using of ontologies and Semantic Web technologies to facilitate the semantic integration of high-throughput “omics” experimental data.

## Figures and Tables

**Table 1:** Commonly used molecular biology databases for functional analysis of gene and protein expression data.

Database name	Database content	Data access and analysis support	URL
*Protein Sequence*			

UniProtKB/Swiss-Prot and UniProtKB/TrEMBL, UniProt Archive (UniParc) [26]	UniProt protein sequences and functional information, comprehensive and non-redundant database that contains most of the publicly available protein sequences in the world	Text search; Blast sequence similarity search; Sequence alignment; Batch retrieval; Database ID mapping; FTP download	http://www.uniprot.org/

NCBI Reference Sequence (RefSeq) [27]	Non-redundant collection of richly annotated DNA, RNA, and protein sequences	Entrez query access; Searching Nucleotide or Protein; Searching Genome; BLAST; FTP download; Sequence Homology searches and retrieval	http://www.ncbi.nlm.nih.gov/

*Gene and Genome*			

GenBank [28]	Genetic sequence database, an annotated collection of all publicly available DNA sequences databases	Database query; Phylogenetics; Genome Analyses; FTP download	http://www.ncbi.nlm.nih.gov/Genbank/
EMBL [29]			http://www.ebi.ac.uk/embl/
DDBJ [30]			http://www.ddbj.nig.ac.jp/

UniGene [31]	Non-redundant set of eukaryotic gene-oriented clusters of transcript sequences, together with information on protein similarities, gene expression, cDNA clone reagents, and genomic location	Entrez query; Library browse; Digital Differential Display; FTP download	http://www.ncbi.nlm.nih.gov/unigene

FlyBase [32]	*Drosophila* sequences and genomic information	Aberration Maps; Batch download; BLAST; Chromosome Maps; Coordinate Converter; CytoSearch; GBrowse; ID Converter; ImageBrowse; Interactions Browser; QueryBuilder; TermLink; FTP download	http://flybase.bio.indiana.edu/

Mouse Genome Database (MGD) [33]	Gene characterization, nomenclature, mapping, gene homologies among mammals, sequence links, phenotypes, allelic variants and mutants, and strain data	Genes & Markers Query; Sequence Query; MouseBLAST; Graphical Map Tools; Mouse Genome Browser; Batch Query; MGI Web Service	http://www.informatics.jax.org/

Saccharomyces Genome Database (SGD) [34]	Genetic and molecular biological information about* Saccharomyces cerevisiae *	Search Gene function information and Protein information; Specialized Gene and Sequence Searches; Search Yeast Literature; BLAST; Batch download; Pattern Matching; Genome Restriction Analysis; PDB Homology Query; Yeast Protein Motif Query; Yeast Biochemical Pathways; Gene Expression Connection	http://www.yeastgenome.org/

WormBase [35]	Data repository for *C. elegans* and *C. briggsae *	Gene, Phenotype, protein, and Genetics Search; Microarray Expression download and Pattern search; Ontology Search	http://www.wormbase.org/

The Arabidopsis Information Resource (TAIR) [36]	The genetic and molecular biology information resource about *Arabidopsis *	Synteny Viewer; MapViewer; Pattern Matching; Motif Analysis; Bulk Data Retrieval; Chromosome Map Tool; Restriction Analysis	http://www.arabidopsis.org/

*Taxonomy*			

NCBI Taxonomy [37]	Names of all organisms that are represented in the genetic databases with at least one nucleotide or protein sequence	Browse; Retrieve and FTP download	http://www.ncbi.nlm.nih.gov/Taxonomy/

UniProt Taxonomy [26]	UniProt taxonomy database, which integrates taxonomy data compiled in the NCBI database and data specific to the UniProt Knowledgebase	Query the database by keywords (species name) or NCBI taxonomic identifier	http://www.uniprot.org/taxonomy/

*Gene Expression*			

Gene Expression Omnibus (GEO) [38]	Public repository for high-throughput microarray experimental data	Search by accession number; Search Entrez GEO DataSets or Entrez GEO Profiles with keywords; Visualize cluster heat map images; Retrieve other genes with similar expression patterns; Retrieve chromosomally closest 20 genes; FTP download	http://www.ncbi.nlm.nih.gov/geo/

CleanEx [39]	Expression reference database that facilitates joint analysis and cross-dataset comparisons	Search by ID, Gene symbol and target ID; List expression datasets; Text search in expression datasets description lines; Extract all features of common genes between datasets; Experiments pools comparison; Batch retrieval; FTP download	http://www.cleanex.isb-sib.ch/

SOURCE [40]	Functional genomics resource for human, mouse and rat to facilitate the analysis of large sets of data using genome-scale experimental approaches	Search by CloneID, Database Accession, Gene name/Symbol, UniGene ClusterID, Probe ID, and Entrez GeneID; Batch retrieval	http://source.stanford.edu/

ArrayExpress [41]	Public repository for well-annotated data from array based platforms, including gene expression, comparative genomic hybridization (CGH) and chromatin-immunoprecipitation (ChIP) experiments, tiling arrays, and so forth	Web-based query interface; REST and Web-services access; FTP download; Web-based online microarray analysis tool—Expression Profiler	http://www.ebi.ac.uk/microarray-as/ae

*Proteomic Peptide ID Databases*			

Global Proteome Machine Database (GPMDB) [42]	Global Proteome Machine Database, which utilizes the information obtained by GPM servers to aid in peptide validation as well as protein coverage patterns	Search by protein description keywords, and data set keywords	http://gpmdb.thegpm.org/

PRoteomics IDEntifications Database (PRIDE) [43]	PRIDE database provides public data repository for proteomics data	Search by PRIDE Experiment accession number and Protein accessions; Browse experiments by project name or categories such as species, tissue, cell type, GO terms and disease; Ontology Lookup Service (OLS); Protein Identifier Cross Reference (PICR) service; Database on Demand (DOD)	http://www.ebi.ac.uk/pride/

Peptidome [44]	Public repository that archives and freely distributes tandem mass spectrometry peptide and protein identification data	Search by Accession, Author, Description, MeSH Terms, Organism, Peptide Count, Platform, Protein Count, Protein GI, Publication Date, Search Engine, Spectra Count, Submitter Institute, Title, Update Date	http://www.ncbi.nlm.nih.gov/peptidome

PeptideAtlas [45]	Peptide database identified by Tandem Mass Proteomics experiments	Search by Protein/Gene Name, Protein/Gene ID, Protein/Gene Symbol, Accession, Refseq, Sequence and Peptide Accession; Browse Peptides; Browse Proteins; FTP download	http://www.peptideatlas.org/

*Protein Expression*			

Swiss-2DPAGE [46]	Annotated 2D gel electrophoresis database contains data on proteins identified on various 2D PAGE and SDS-PAGE reference maps	Search by description, accession number, author, spot serial number, experimental pI/Mw range and experimental identification methods; Retrieve all the protein entries identified on a given reference map; Compute estimated location on reference maps for a user-entered sequence; FTP download	http://ca.expasy.org/ch2d

*Function and Pathway*			

Kyoto Encyclopedia of Genes and Genomes (KEGG) [47]	Integrated database resource consisting of 16 main databases, broadly categorized into systems information, genomic information, and chemical information	Access by KEGG object identifier; KEGG Web Services and KEGG FTP download; Pathway Mapping; Brite Mapping; KegHier for browsing and searching functional hierarchies in KEGG BRITE; KegArray for analysis of transcriptome data (gene expression profiles) and metabolome data (compound profiles)	http://www.genome.jp/kegg/

BioCyc [48]	Microbial pathway/genome databases	Visualize individual metabolic pathways; View the complete metabolic map of an organism; Genome browsing capabilities and comparative analysis tools	http://biocyc.org/

*Genetic Variation and Disease*			

Online Mendelian Inheritance in Man (OMIM) [49]	A catalog of human genetic and genomic phenotypes	Entrez search at basic, advanced, or complex Boolean levels; Browse entries; Build query; Combine search results; Store search results in Clipboard; FTP download	www.ncbi.nlm.nih.gov/sites/entrez?db=omim

HapMap [50]	Resource for human genetic variation	Browse data; Bulk data download; HapMart—a data mining tool for retrieving data from the HapMap database	http://www.hapmap.org/

*Ontology*			

Gene Ontology (GO) [51]	Gene Ontology database provides controlled vocabulary of terms describing Biological process, Cellular component, and Molecular function of gene and gene product annotation data	Tools include Browsers, Microarray tools, Annotation tools, Mapping to other databases, FTP download in Flat file, MySQL or RDF XML format	http://www.geneontology.org/

*Interaction*			

IntAct [52]	Protein-protein interaction data	Browse by UniProt Taxonomy, Gene Ontology, Interpro Domain, Reactome Pathway, Chromosomal Location, and mRNA expression, FTP download in PSI-MI and PSI-MI TAB format	http://www.ebi.ac.uk/intact

Database of Interacting Proteins (DIP) [53]	Database of experimentally determined interactions between proteins with curator or computational methods generated annotations	Search by protein entry, BLAST, Motif, Article and pathBLAST; Data analysis services include Expression Profile Reliability Index, Paralogous Verification, and Domain Pair Verification	http://dip.doe-mbi.ucla.edu/

*Modification*			

RESID [54]	Collection of annotations and structures for Protein Pre-, Co- and Post-translational modifications	Web-based search interface; FTP download database entries in XML format, and associated files containing XML DTD, graphic images, and molecular models	http://www.ebi.ac.uk/RESID

Phosphosite [55]	Database of phosphorylation sites and other Post-translational modifications	Search by Protein, Sequence, or Reference; Browse MS data by Disease, Cell Line, and Tissue	http://www.phosphosite.org/

*Structure*			

Protein Data Bank (PDB) [56]	Database of experimentally-determined structures of proteins, nucleic acids, and complex assemblies	Web-based search and browsing interface; File download via http and FTP services in PDB, mmCIF, and PDBML/XML format	http://www.pdb.org/pdb/home/home.do

Structural Classification of Proteins (SCOP) [57]	Comprehensive ordering of all proteins of known structure according to their evolutionary and structural relationships	Keywords-based search	http://scop.mrc-lmb.cam.ac.uk/

CATH [58]	Protein domain structures database	Search by ID/Keywords and FASTA sequence; BLAST; Cathedral server, and SSAP server for query and analysis CATH data; FTP download	http://www.cathdb.info/

Molecular Modeling Database (MMDB) [59]	Database of 3D structures	Search by UID/text term, protein sequence and 3D coordinates; FTP download	http://www.ncbi.nlm.nih.gov/Structure/MMDB/mmdb.shtml

PDBsum [60]	Summaries and analyses of PDB structures	Search by text or sequence; Browse by Highlights, List of PDB codes, Het Groups, Ligands, Enzymes, ProSite and Species; Download data file for protein names, protein sequences, protein annotations, Enzymes, Het Groups, and Ligands	http://www.ebi.ac.uk/pdbsum

Protein Structure Model Database (Modbase) [61]	Annotated comparative protein structure models and related resources	Search by model or sequence similarity and properties	http://modbase.compbio.ucsf.edu/modbase-cgi/index.cgi

*Classification*			

PIRSF [62]	Family/superfamily classification of whole proteins	Batch retrieval using UniProtKB AC, PIRSF ID, Pfam ID, COG ID, EC Number, GO ID, KEGG Pathway ID, PDB ID; PIRSF scan by sequence or UniProtKB identifier; FTP download	http://pir.georgetown.edu/pirwww/dbinfo/pirsf.shtml

UniProt Reference Clusters (UniRef) [26]	UniProt non-redundant reference clusters	Searches on various attributes of the UniRef clusters, including UniRef cluster ID, protein names, organism names and database identifiers; Direct web access in HTML, XML and FASTA format; FTP download in XML format	http://www.uniprot.org/help/uniref

Pfam [63]	Protein families of domains each represented by multiple sequence alignments and hidden Markov models (HMMs)	Search by Sequence, Functional similarity, Keyword, Domain, DNA, and Taxonomy; Browse by Families, Clans, Proteomics; FTP download	http://pfam.sanger.ac.uk

InterPro [64]	Integrated resource of protein families, domains, and functional sites	Text search; SRS text search; InterPro Scan; InterPro BoMart; Web services; FTP download	http://www.ebi.ac.uk/interpro

Protein ANalysis THrough Evolutionary Relationships (PANTHER) Classification System [65]	Gene products organized by biological function	Search; Browse; Batch search; Gene expression data analysis; Evolutionary analysis of coding SNPs; HMM sequence scoring; FTP download	http://www.pantherdb.org/panther

Simple Modular Architecture Research Tool (SMART) [66]	Resource for protein domain identification and the analysis of protein domain architectures	Sequence analysis; Architecture analysis; Domain detection	http://smart.embl-heidelberg.de/

**Table 2:** Database identifiers supported by PIR ID mapping service.

From	To
*Sequence*	*Sequence*
FLY ID, GenBank AC, Genpept AC, GI Number, IPI ID, MGI	FLY ID, GenBank AC, Genpept AC, GI Number, IPI ID, MGI
ID, NREF ID, PIR-PSD ID, PIR-PSD AC, Refseq AC, SGD ID,	ID, NREF ID, PIR-PSD ID, PIR-PSD AC, Refseq AC, SGD ID,
TIGR ID, UniParc AC, UniProtKB AC, UniProtKB ID	TIGR ID, UniParc AC, UniProtKB AC, UniProtKB ID,
	UniRef50, UniRef90, UniRef100

*Classification*	*Classification *
BLOCKS ID, COG ID, Pfam ID, PIRSF ID, PRINTS ID, PROSITE	BLOCKS ID, COG ID, Pfam ID, PIRSF ID, PRINTS ID,
ID, UniRef50, UniRef90, UniRef100	PROSITE ID

*Function/Feature *	*Function/Feature *
BIND ID, EC Number, GO ID, KEGG Pathway ID, RESID ID	BIND ID, EC Number, GO ID, KEGG Pathway ID, RESID ID

*Organism *	*Organism *
Taxon Group ID, Taxon ID	Taxon Group ID, Taxon ID

*Miscellaneous*	*Miscellaneous*
Entrez Gene ID, OMIM ID, PDB ID, PubMed ID, Gene Name	Entrez Gene ID, OMIM ID, PDB ID, PubMed ID, Gene Name

**Table 3:** NIAID biodefense proteomics resource catalog summary.

Organism	PRC	Data Type	SOPs	No. of protein in Master Protein Directory (MPD)	No. of reagents in Master Reagent Directory (MRD)	No. of proteins in Complete Predicated Proteome (CPP)
*Brucella abortus*	Caprion Proteomics Inc.	Mass spectrometry	23	4963	—	6070
*Cryptosporidium parvum*	Einstein Biodefense Proteomic Research Center	Mass spectrometry	4	609	Antibodies (68)	—
*Francisella tularensis*	Myriad Genetics	Protein interaction	4	62	Clone (4379)	4629
*Monkeypox*	Pacific Northwest National Laboratory	Mass spectrometry	2	2958	Antibodies (1)	187
*SARS-CoV*	Scripps Research Institute	Protein structure	5	6	Clone (7)	—
*Toxoplasma gondii*	Einstein Biodefense Proteomic Research Center	Mass spectrometry	5	6678	Antibodies (101)	—
*Vaccinia*	Myriad Genetics	Protein interaction	2	33	Clone (315)	254
	Pacific Northwest National Laboratory	Mass spectrometry	2	2973	—	—
*Vibrio cholera*	Harvard Institute of Proteomics	Clone	4	3731	Bacteria (627) Clone (7172)	11208
*Yersinia pestis*	Myriad Genetics	Protein interaction	5	75	Clone (9900)	5966
*Bacillus anthracis*	Harvard Institute of Proteomics	Clone	3	5342	Clone (5344)	
	University of Michigan	Mass spectrometry	—	5851	Bacteria (22) ArrayChip (1)	
						16686
	University of Michigan	Microarray	2	6378	—	
	Myriad Genetics	Protein interaction	5	84	Clone (7884)	
*Salmonella typhi*	Pacific Northwest National Laboratory	Mass spectrometry	—	2061	Bacteria (38)	—
*Salmonella typhimurium*	Pacific Northwest National Laboratory	Protein interaction	—	3	—	
	Pacific Northwest National Laboratory	Mass spectrometry	12	3753	—	4532
	Pacific Northwest National Laboratory	Microarray	—	653	—	
